# Yellow Fever Virus DNA in Urine and Semen of Convalescent Patient, Brazil

**DOI:** 10.3201/eid2401.171310

**Published:** 2018-01

**Authors:** Carla M. Barbosa, Nicholas Di Paola, Marielton P. Cunha, Mônica J. Rodrigues-Jesus, Danielle B. Araujo, Vanessa B. Silveira, Fabyano B. Leal, Flávio S. Mesquita, Viviane F. Botosso, Paolo M.A. Zanotto, Edison L. Durigon, Marcos V. Silva, Danielle B.L. Oliveira

**Affiliations:** University of São Paulo, São Paulo, Brazil (C.M. Barbosa, N. Di Paolo, M.P. Cunha, M.J. Rodrigues-Jesus, D.B. Araujo, V.B. Silveira, F.B. Leal, F.S. Mesquita, P.M.A. Zanotto, E.L. Durigon, D.B.L. Oliveira);; Butantan Institute, São Paulo (V.F. Botosso); Institute of Infectology Emilio Ribas, São Paulo (M.V. Silva);; Pontifical Catholic University, São Paulo (M.V. Silva)

**Keywords:** yellow fever, yellow fever virus, YFV, viruses, virus diagnostics, flavivirus, vector-borne infections, urine, semen, convalescent patient, Brazil

## Abstract

Yellow fever virus RNA is usually detected in blood of infected humans. We detected virus RNA in urine and semen samples from a convalescent patient. A complete virus genome was sequenced for an isolate from a urine sample. This virus had a South American I genotype and unique synapomorphic changes.

Yellow fever virus (YFV) is a member of the genus *Flavivirus* and causes yellow fever in humans, characterized by fever, prostration, and hepatic, renal, and myocardial complications that lead to death in 20%–50% of cases (*1*). Clinical confirmation of YFV infections is based on detection of virus RNA in blood by reverse transcription PCR or antigen-based ELISAs. Detection of virus in urine samples has been used for confirming infections with flaviviruses, including West Nile virus (*2*), Zika virus (*3*), dengue virus (*4*), and YFV (*5*).

Despite availability of an effective vaccine, >200,000 cases of yellow fever and >30,000 deaths occur per year (*6*). A large epidemic of yellow fever with high death rates recently occurred in Brazil. In December 2016, the first cases of yellow fever during this epidemic were reported in Minas Gerais; cases were later identified in Espírito Santo, Goiás, Mato Grosso, Pará, Rio de Janeiro, São Paulo, Tocantins, and the Federal District. There were 792 confirmed cases and 274 deaths (case-fatality rate 35%) as of July 10, 2017 (*7*). We report a case of yellow fever in a 65-year-old man who was a native of São Paulo and had not been vaccinated against yellow fever. The study protocol was approved by the Ethics Committee on Research with Human Beings at the University of Sao Paulo. The patient provided informed consent for use of the samples during the study.

The patient had traveled to Januária, Minas Gerais, Brazil, on December 28, 2016, and to a rural area north of São Paulo on January 3, 2017. On January 6, he had fever, chills, body pain, and nausea. During days 1–3 after symptom onset, more severe symptoms developed: persistent fever (temperature 39.5°C–40°C), headache, body pain, prostration, vomiting, dizziness, anorexia, dark stools, dark yellow urine, and bitterness in the mouth.

The patient was admitted to a public hospital in Januária on January 9. An ELISA for nonstructural protein 1 (NS1) of dengue virus showed a negative result. The patient also had severe thrombocytopenia (platelet count 77,000/mm^3^ [reference range 140,000–450,000/mm^3^]).

On January 13, the patient returned to São Paulo and was admitted to a public hospital. Another ELISA for dengue virus NS1 was performed and showed a negative result. His platelet count decreased to 57,000/mm^3^. On January 16, the patient was admitted to a reference hospital for infectious diseases in São Paulo. He showed a moderate clinical presentation: anicteric form and mild spontaneous hemorrhage (ecchymosis in the right eye). High fever, gastrointestinal symptoms (vomiting and diarrhea), weakness, adynamia, and generalized myalgia were also observed. The patient had a weight loss of 4 kg over 8 days. Serum and urine samples were obtained (Figure, panel A; Technical Appendix).

**Figure:**
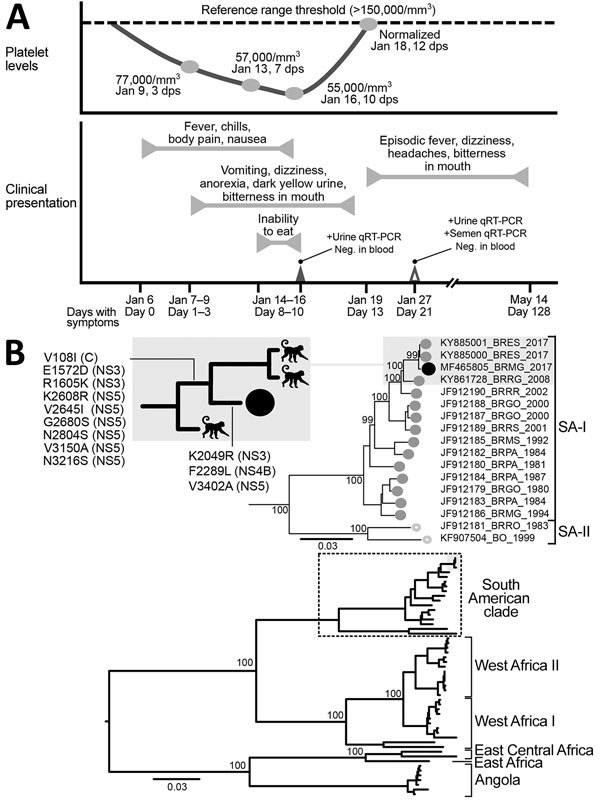
Clinical progression and detection of YFV RNA in urine and semen of convalescent patient, Brazil. A) Platelet levels, clinical parameters and symptoms, and test results over a 128-day period after initial symptoms were observed. B) Maximum-likelihood tree (midpoint-rooted) inferred by using complete genomes of YFV to distinguish major virus genotypes; dashed box indicates South American clade strains, enlarged at top. Numbers near nodes indicate percent bootstrap values after 10,000 replicates for major branches. Black circles indicate virus isolated in this study. Shaded boxes indicate monkey-derived virus sister taxa sampled during the same outbreak; inset at top left shows most parsimonious reconstructions of synapomorphic changes detected NS3, NS4B, and NS5 genes. GenBank accession number, geographic location code, and year of isolation are shown for virus isolates. Scale bars indicate nucleotide substitutions per site. C, capsid; dps, days postsymptom onset; Neg., negative; NS, nonstructural; qRT-PCR, quantitative reverse transcription PCR; SA, South America clade; YFV, yellow fever virus; +, positive.

We extracted virus RNA by using the NucliSENS EasyMag Kit (bioMérieux, Marcy l’Étoile, France). We tested samples for YFV RNA by using a real-time quantitiative reverse transcription PCR (qRT-PCR) and primers specific for YFV (*8*) and a conventional PCR and pan flavivirus primers (*9*). Serum samples showed negative results for both PCRs. However, a urine sample obtained 10 days after initial symptoms was positive for YFV RNA (cycle threshold [C_t_] 17.42, 9.3 × 10^6^ RNA copies/mL) by qRT-PCR. We also performed a qualitative IgM-capture ELISA with a specific virus antigen and obtained positive results (optical density 1.19) (*10*).

On January 27, we obtained serum, urine, and semen samples and tested them by using qRT-PCR. Urine (C_t_ 28.57, 3.3 × 10^3^ RNA copies/mL) and semen (C_t_ 31.00, 5 × 10^2^ RNA copies/mL) samples were positive for YFV RNA. To evaluate infectivity, we tested a urine sample obtained on this date (Technical Appendix). We isolated YFV in cell culture, which confirmed virus integrity. We also confirmed infectivity after a second virus passage (C_t_ 24.35, 6.7 × 10^4^ RNA copies/mL).

For the urine sample that was positive for YFV RNA by qRT-PCR, we directly characterized viral diversity by using next-generation sequencing (Technical Appendix). In South America, phylogenetic studies have inferred 2 circulating YFV genotypes. The isolate from our patient (BRMG-2017) clustered with South America I isolates, including 2 viruses isolated in 2017 in Espírito Santo, a state bordering Minas Gerais, and other viruses isolated previously in Brazil (Figure, panel B).

We did not observe any insertions or deletions in BRMG-2017 nucleotide sequences when compared with sequences of other South America I strains. However, several synapomorphic changes were detected (V108I [capsid], E1572D [NS3], R1605K [NS3], K2608R [NS5], V2645I [NS5], G2680S [NS5], N2804S [NS5], V3150A [NS5], and N3216S [NS5]). Most of these changes were located in the NS5 (RNA-dependent RNA polymerase) gene, which plays a major role in virus replication. Changes in the NS5 gene have been associated with differences in viral replication, immune response, and protein–protein interactions during virus replication.

Our results suggest that semen can be a useful clinical material for diagnosis of yellow fever and indicate the need for testing urine and semen samples from patients with advanced disease. Such testing could improve diagnostics, reduce false-negative results, and strengthen the reliability of epidemiologic data during ongoing and future outbreaks.

Technical Appendix Additional information on yellow fever virus in urine and semen of convalescent patient, Brazil.
