# Supplementary material for: Yellow Fever Virus DNA in Urine and Semen of Convalescent Patient, Brazil
Source: Emerg Infect Dis. 2018 Jan;24(1):176–8. doi: 10.3201/eid2401.171310 (PMC5749440; doi:10.3201/eid2401.171310)
Supplement: Technical Appendix — Additional information on yellow fever virus in urine and semen of convalescent patient, Brazil. [file 17-1310-Techapp-s1.pdf]

Article DOI: https://doi.org/10.3201/eid2401.171310

Yellow Fever Virus in Urine and Semen of Convalescent Patient, Brazil

Technical Appendix

Clinical Findings

The case-patient showed a moderate clinical presentation of yellow fever. He had fever, headache, body pain, anorexia, dizziness, bitterness in the mouth, and dark yellow urine. The patient was no longer able to eat solid food during a 3-day period. Initial laboratory test results showed a platelet count 55,000/mm^3; basophil count 100 cells/mm^3 (reference range 0.0–0.2 cells/mm^3); alanine aminotransferase 343 U/L (reference range 0–55 U/L); aspartate aminotransferase 156 U/L (reference range 5–34 U/L); total bilirubin 1.11 mg/dL (reference range 0.2–1.2 mg/dL); creatine phosphokinase 157 U/L (reference value ≤170 U/L), creatinine 1 mg/dL (reference range 15–55 mg/dL); prothrombin time 13.4 s (reference range 10.7–15.0 s); urea 37 mg/dL (reference range 0.72–1.25 mg/dL); prothrombin activity 101% (reference value >70%); and C-reactive protein 0.90 mg/L (reference range 0.00–5.00 mg/dL).

Liver ultrasonography showed a moderate degree of fatty liver infiltration. Doppler imaging showed normal portal vein flow. On day 11 post-onset of symptoms (dps 11) he started eating solid food again and his urine showed a standard color. Normalization of his platelet count was observe on dps 12, and aminotransferase levels within reference ranges were observed on dps 39. During dps 13–dps 76, the patient reported having episodes of fever, dizziness, headaches, and bitterness in the mouth, especially during physical excursion. He also reported a loss of taste.

Next-Generation Sequencing

To obtain the complete genome sequence, virus RNA was reextracted from the urine sample by using the QIAamp Viral RNA Mini Kit (QIAGEN, Valencia, CA, USA), purified with DNase I, and concentrated by using the RNA Clean and Concentrator-5 Kit (Zymo Research; Irvine, CA, USA). Paired-end RNA libraries were constructed and validated by using the TruSeq Stranded Total RNA HT Sample Prep Kit (Illumina; San Diego, CA). Sequencing was

performed on an Illumina NextSeq platform at Core Facility for Scientific Research at the University of São Paulo. Low-quality and unpaired reads/bases were filtered by using Trimmomatic version 0.36 (*1*). Paired-end reads ($Q_{phred>33}$) were aligned to the reference sequence (Genbank accession no. KY885001) by using Bowtie2 (*2*). Using Samtools version 0.1.18 (http://samtools.sourceforge.net/), we extracted a consensus sequence of 10,948 nt and a 112.6× average depth and obtained a 99.5% breadth of coverage of the reference genome, with bases lacking only at the extremes of the untranslated region.

To infer the phylogenetic relationships of the yellow fever virus (YFV) sequence (GenBank accession no. MF465805) with other YFV sequences, we downloaded all available complete genome sequences from GenBank. We excluded all vaccine-derived strains except for the 17D viscerotropic strain isolated in 1975 (*3*). Sequences were aligned by using Clustal-Omega version 1.2.1 (http://www.clustal.org/omega/) and manually curated by using JalView version 1.18-β8 (http://www.jalview.org/download). A maximum-likelihood tree was estimated in the nucleotide substitution general time-reversible with gamma-distributed rate variation and invariant sites model. Support for the tree was accessed after 10,000 nonparametric bootstrap replicates with FastTree version 2.1.8 (https://bioweb.pasteur.fr/packages/pack@FastTree@2.1.8).

Virus isolation in Cell Culture

We attempted to isolate YFV from the positive urine sample on day 21 after onset of patient symptoms to confirm the presence of infectious virus. The sample was initially treated with 100× Antibiotic-Antimycotic (Gibco, Gaithersburg, MD, USA) for 1 h on ice. For virus isolation, a Vero-E6 cell culture was maintained in 25-cm^2 flakes by using high-glycoside Dulbecco's modified Eagle's medium supplemented with 10% fetal bovine serum (Gibco), 1% nonessential amino acids (Gibco), and 1% sodium pyruvate (Gibco) and maintained at 37°C in an atmosphere of 5% CO_2. After reaching ≈90% confluence in the monolayer, we prepared a 1:3 mixture with pure Dulbecco's modified Eagle's medium (pH 8.2), inoculated the mixture into Vero-E6 cells, and incubated the mixture for 1 h with gentle shaking every 10 min to enable homogeneous adsorption of viruses. At the end of the adsorption period, 5 mL of the culture medium (pH 7.2), plus 2% fetal bovine serum, 1% nonessential amino acids, and 1% sodium pyruvate were added. The sample was passed 2 times in cell culture and after 7 days of

incubation, cells were collected and tested for YFV by using a real-time quantitative reverse transcription PCR and a standard PCR.

References

1. Bolger AM, Lohse M, Usadel B. Trimmomatic: a flexible trimmer for Illumina sequence data. Bioinformatics. 2014;30:2114–20. PubMed http://dx.doi.org/10.1093/bioinformatics/btu170

2. Langmead B, Salzberg SL. Fast gapped-read alignment with Bowtie 2. Nat Methods. 2012;9:357–9. PubMed http://dx.doi.org/10.1038/nmeth.1923

3. Engel AR, Vasconcelos PF, McArthur MA, Barrett AD. Characterization of a viscerotropic yellow fever vaccine variant from a patient in Brazil. Vaccine. 2006;24:2803–9. PubMed http://dx.doi.org/10.1016/j.vaccine.2006.01.009
